# The Combined Dexamethasone/CRH Test (DEX/CRH Test) and Prediction of Acute Treatment Response in Major Depression

**DOI:** 10.1371/journal.pone.0004324

**Published:** 2009-01-29

**Authors:** Cornelius Schüle, Thomas C. Baghai, Daniela Eser, Sibylle Häfner, Christoph Born, Sascha Herrmann, Rainer Rupprecht

**Affiliations:** Department of Psychiatry and Psychotherapy, Ludwig-Maximilian-University of Munich, Munich, Germany; James Cook University, Australia

## Abstract

**Background:**

In this study the predictive value of the combined dexamethasone/CRH test (DEX/CRH test) for acute antidepressant response was investigated.

**Methodology/Principal Findings:**

In 114 depressed inpatients suffering from unipolar or bipolar depression (sample 1) the DEX/CRH test was performed at admission and shortly before discharge. During their stay in the hospital patients received different antidepressant treatment regimens. At admission, the rate of nonsuppression (basal cortisol levels >75.3 nmol/l) was 24.6% and was not related to the later therapeutic response. Moreover, 45 out of 114 (39.5%) patients showed an enhancement of HPA axis function at discharge in spite of clinical improvement. In a second sample, 40 depressed patients were treated either with reboxetine or mirtazapine for 5 weeks. The DEX/CRH test was performed before, after 1 week, and after 5 weeks of pharmacotherapy. Attenuation of HPA axis activity after 1 week was associated with a more pronounced alleviation of depressive symptoms after 5-week mirtazapine treatment, whereas downregulation of HPA system activity after 5 weeks was related to clinical response to reboxetine. However, early improvement of HPA axis dysregulation was not necessarily followed by a beneficial treatment outcome.

**Conclusions/Significance:**

Taken together, performance of a single DEX/CRH test does not predict the therapeutic response. The best predictor for response seems to be an early attenuation of HPA axis activity within 1 or 2 weeks. However, early improvement of HPA system dysfunction is not a sufficient condition for a favourable response. Since a substantial part of depressive patients display a persistence of HPA axis hyperactivity at discharge, downregulation of HPA system function is not a necessary condition for acute clinical improvement either. Our data underline the importance of HPA axis dysregulation for treatment outcome in major depression, although restoration of HPA system dysfunction seems to be neither a necessary nor a sufficient determinant for acute treatment response.

## Introduction

According to the corticosteroid-receptor hypothesis of depression hypothalamic-pituitary-adrenocortical (HPA) system dysregulation plays an important role in the pathophysiology of depression [1], [2]. In depressed patients, elevated cortisol (COR) and adrenocorticotrophic hormone (ACTH) concentrations in the plasma [3]–[6] or in the cerebrospinal fluid (CSF) [7] have been found. Additionally, HPA axis hyperactivity is obviously reflected by elevated urinary free cortisol (UFC) levels, which appear to be approximately twofold higher in depressed patients as compared to healthy controls [8]. Further investigations using neuroendocrine challenge tests confirmed the hypothesis of a profound HPA axis dysregulation in depression: Several studies using the corticotropin-releasing hormone (CRH)-stimulation test reported a blunted ACTH response whereas the COR stimulation was indistinguishable from normal controls [9], [10]. In contrast to a reduced ACTH response to CRH, depressive patients show both an enlargement of the adrenal gland [11], [12] and elevated COR stimulation patterns indicating an enhanced adrenal sensitivity after challenge with ACTH in most [13]–[15] but not all [16] studies. Findings in depressed patients of increased CRH levels in the CSF [17] and elevated numbers of CRH [18] and arginine-vasopressin (AVP) [19] expressing neurons in the paraventricular nucleus of the hypothalamus as well as the observation of reduced CRH binding sites in the frontal cortex of suicide victims [20] gave reason to the assumption that depression is characterized by a hypothalamic overdrive of CRH and/or AVP which in consequence leads to receptor down-regulation in the corticotrophs of the pituitary gland.

Moreover, it has been suggested that an impaired signalling pathway via corticosteroid-activated mineralocorticoid and glucocorticoid receptors, leading to an impaired negative feedback regulation of the HPA system, causes this hyperactivity [21]. With regard to the glucocorticoid receptor, a disturbed negative feedback control in depressed patients is reflected by COR escape from dexamethasone suppression [22], [23] as well as an increased ACTH and COR release in the combined dexamethasone-suppression/CRH-stimulation test (DEX/CRH test) [24], [25]. The DEX/CRH test at present is considered to be the most sensitive tool to demonstrate a disturbed regulation of the HPA axis in depressed patients and has been shown to have a sensitivity of more than 80% if subjects are clustered into different age ranges [25].

A gradual down-regulation of HPA axis hyperactivity in depressed patients as measured by serial DEX/CRH tests has been demonstrated for tricyclic antidepressants such as amitriptyline [26], doxepin [27], trimipramine [28]–[31], for the selective serotonin-reuptake inhibitor paroxetine [32], for tianeptine which enhances the presynaptic reuptake of serotonin [32], and for the selective norepinephrine reuptake inhibitor reboxetine [33]. Proponents of the corticosteroid receptor hypothesis of depression emphasize that a gradual normalization of HPA system dysregulation as measured by the DEX/CRH test precedes or coincides with the response to antidepressant treatment and is a necessary prerequisite for clinical remission to become manifest, whereas persisting COR hypersecretion during the DEX/CRH test at discharge in spite of clinical improvement may be an indicator for an enhanced risk for relapse within the following six months [34], [35]. In addition, in outpatients with clinically remitted major depression, higher cortisol levels in the DEX/CRH test are apparently associated with relapse of major depression [36], [37]. Interestingly, persisting nonsuppression in the single dexamethasone suppression test (DST) also indicates a higher risk for relapse within the following months [38]–[43]. It has further been postulated that antidepressants may exert their therapeutic effects at least partly through their actions on the HPA system and that all antidepressants developed so far may have a uniform dampening impact on HPA axis function irrespective of their type of action within monoaminergic systems [1], [2], [44]–[46].

The present study aims to answer the following questions:

- What is the nonsuppression rate in the DEX/CRH test in acutely depressed inpatients within the first week after admission to a psychiatric hospital before starting antidepressant therapy?
- Is the normalization of HPA axis hyperactivity during antidepressant therapy (as measured by serial DEX/CRH tests) a **necessary condition** for clinical response? Are there depressed patients who respond to antidepressant therapy although their HPA axis hyperactivity is not attenuated or even further increased?
- Is the normalization of HPA axis hyperactivity during antidepressant treatment a **sufficient condition** for clinical response? Are there depressed patients who do not respond to subsequent antidepressant therapy in spite of early improvement of HPA axis dysregulation?
- Is the DEX/CRH test at admission or its change during antidepressant treatment suitable for prediction of acute therapeutic response?

## Materials and Methods

### Sample 1

114 depressed inpatients (53 men, 61 women) aged between 18 and 74 years (mean age 46.48±13.81 years) entered the study after the procedures had been fully explained and written informed consent had been obtained. The patients were diagnosed by experienced and trained psychiatrists according to DSM-IV criteria [47] using the Structured Clinical Interview for DSM-IV, German version [48]. Inclusion criteria for the depressed patients were a) a major depressive episode with melancholic features, according to DSM-IV criteria (DSM-IV: 296.2, 296.3) or bipolar depression (DSM-IV: 296.5) b) a sum score of at least 18 on the 17-item version of the Hamilton Depression Rating Scale (17-HAMD) [49] c) exclusion of major medical disorders; availability of normal laboratory parameters; normal blood pressure; normal electrocardiogram; and normal encephalogram d) exclusion of addiction or other comorbid psychiatric diagnoses e) no psychotropic drugs for at least 3 days before the first DEX/CRH test with the exception of zopiclone (up to 7.5 mg per day) in case of sleep difficulties and lorazepam (up to 2 mg per day) in case of inner tension and anxiety f) exclusion of pregnancy or use of oral contraceptives g) no use of oral steroid hormones or hormonal replacement therapy which may influence the results of the DEX/CRH test. Further clinical characteristics are given in **Table 1**.

**Table 1:** Demographic and clinical parameters in 114 inpatients suffering from unipolar or bipolar depression who underwent the DEX/CRH test at admission and at discharge (sample 1).

	all patients	suppressors	nonsuppressors	Statistics	COR peak Im	COR peak NIm	Statistics
	(n = 114)	test1 (n = 86)	test1 (n = 28)	suppression	test2 (n = 69)	test2 (n = 45)	COR peak improvement
diagnoses							
MD, first episode	35	25	10		26	9	
MD, recurrent	72	56	16	χ2 = 0.577; p = 0.749	39	33	χ2 = 5.026; p = 0.134
bipolar depression	7	5	2		4	3	
gender (M/F)	53/61	44/42	9/19	χ2 = 3.072; p = 0.080	34/35	19/26	χ2 = 0.545; p = 0.461
age	46.5±13.8	46.4±13.5	46.8±15.0	F = 0.014; p = 0.907	47.5±12.8	44.9±15.3	F = 0.990; p = 0.322
height	171.0±13.0	170.7±14.3	171.9±8.0	F = 0.178; p = 0.674	171.3±8.3	170.6±18.1	F = 0.079; p = 0.779
BMI	28.7±4.2	30.1±4.8	24.3±3.8	F = 0.407; p = 0.525	25.0±4.5	34.4±66.6	F = 1.378; p = 0.243
age of onset	38.0±12.8	38.2±13.3	37.1±11.2	F = 0.166; p = 0.685	40.1±11.6	34.7±14.0	**F = 5.121; p = 0.026**
number of depressive episodes	3.47±3.77	3.42±3.89	3.64±3.45	F = 0.074; p = 0.786	3.19±3.33	3.91±4.37	F = 0.999; p = 0.320
duration of inpatient status	64.6±37.2	66.6±40.7	58.5±22.8	F = 1.008; p = 0.318	58.4±27.0	74.2±47.8	**F = 5.046; p = 0.027**
17-HAMD sum score at baseline	24.4±5.4	23.8±5.1	26.2±5.8	**F = 4.415; p = 0.038**	24.7±5.5	23.9±5.2	F = 0.520; p = 0.472
response (NRs/Rs)	58/56	44/42	14/14	χ2 = 0.011; p = 0.915	32/37	26/19	χ2 = 1.417; p = 0.234
remission (NRm/Rm)	86/28	64/22	22/6	χ2 = 0.197; p = 0.657	49/20	37/8	χ2 = 1.846; p = 0.174

Mean±standard deviation (SD) is indicated. Patients are subdivided into suppressors/nonsuppressors at admission and into COR peak Im (improvers)/COR peak NIm (nonimprovers) at discharge. Suppressor: COR_1500_ in the DEX/CRH test 1 at admission ≤75.3 nmol/l. COR peak Im ( = COR peak improver): Reduction of the COR peak value in the DEX/CRH test between admission and discharge. MD = Major Depression. M = male, F = female. BMI = body mass index. 17-HAMD = Hamilton Depression Rating Scale, 17-item version. NRs = nonresponders; Rs = responders. NRm = nonremitters; Rm = remitters. Response and remission rates at week 4 are shown. Statistics: results of χ^2^-test or oneway ANOVA are provided. Significant results are given in bold letters.

With regard to the DEX/CRH test procedure, participants received an oral dose of 1.5 mg dexamethasone at 11:00 PM the day before stimulation. On the following day, patients had to rest supine on a bed at 02:00 PM. An intravenous catheter was inserted into the antecubital vein before 02:15 PM and kept open with physiological saline solution. Blood samples were collected at 03:00, 03:15, 03:30, 03:45, 04:00, and 04:15 PM. Each sample was immediately centrifuged and stored at −80° C for COR measurements. At 03:02 PM 100 µg hCRH (Clinalfa AG, Läufelfingen, Switzerland) reconstituted in 1 ml 0.02% HCL in 0.9% saline were injected within 30 sec. For determination of COR serum concentrations, a commercial radioimmunoassay kit was employed (Cortisol-RIA, DPC Biermann, Germany) with a sensitivity of 8.27 nmol/l. Our intra- and interassay coefficients of variation were below 5%. We abstained from reporting the ACTH levels, since COR has been demonstrated to be the best parameter for analyzing DEX/CRH test results and since most established cut-off criterions are related to COR levels but not ACTH concentrations. For the DEX/CRH test the total COR AUC values (total area under the concentration time curve), determined by the trapezoid rule according to Simpson [50], were used for determination of the COR response to the hCRH challenge in the dexamethasone pretreated patients representing the combined effects of altered glucocorticoid receptor (GR) function and hyperdrive of endogenous CRH and vasopressin.

The first DEX/CRH test was administered within the first week after admission to the Department of Psychiatry and Psychotherapy, Ludwig-Maximilian-University of Munich. A wash-out period of 3 days before neuroendocrine testing was mandatory. After the first DEX/CRH test, the patients were treated according to clinical judgement with pharmacological and non-pharmacological antidepressant treatment options at the discretion of the doctor in attendance. Within the following 4 weeks after the first DEX/CRH test, 6 patients were treated with SSRIs, 22 patients with reboxetine, 12 with mirtazapine, 3 with venlafaxine, 3 with tricyclic antidepressants, 6 with antidepressant and lithium augmentation, 2 with antidepressant and anticonvulsant augmentation (not carbamazepine), 29 with pharmacological combination therapies, 8 received electroconvulsive therapy (ECT), and 23 were treated with 2-week transcranial magnetic stimulation (TMS) followed by antidepressant pharmacotherapy. Clinical response was defined by a reduction of at least 50% in the 17-HAMD sum score after 4 weeks of antidepressant treatment. Remission after week 4 was assumed if the 17-HAMD sum score was lower than 9 points. In case of nonresponse, either an augmentation/combination strategy or use of another antidepressant with a different pharmacological profile was initiated. Patients were discharged after they had recovered from the depressive episode. Within the last week before discharge, a second DEX/CRH test was performed.

The dexamethasone suppression status (suppression versus nonsuppression) within the DEX/CRH test at admission was defined by a cut-off criterion of 27.5 ng/ml (∼75.3 nmol/l) applied to the baseline COR level (COR concentration at 03:00 PM, i.e. after administration of 1.5 mg dexamethasone, but immediately before CRH-challenge) which was derived from a normative database from the Max-Planck-Institute of Psychiatry in Munich after correction of a linear bias (Heuser criterion) [25], [51]. Moreover, a further criterion was employed which has been proposed by a Japanese research group [52], [53] defining subjects as nonsuppressors (baseline COR≥50 ng/ml [∼137.95 nmol/l]), intermediate suppressors (baseline COR<50 ng/ml and peak COR≥50 ng/ml), and suppressors (peak COR<50 ng/ml) (Kunugi criterion). HPA axis activity at the time of the DEX/CRH test at discharge was categorized in improvers and nonimprovers according to the change in the peak COR level after CRH challenge between DEX/CRH test 1 (admission) and test 2 (discharge). A COR peak improver was defined by a lower COR peak concentration during test 2; otherwise, a COR peak nonimprover was presumed. The peak COR level was used for the categorization into HPA system improvers and nonimprovers instead of the COR AUC value to be in line with previous definitions of HPA system improvement in remitted depression [34], [35], [54].

### Sample 2

Data of the second patient sample have been already reported in a previous publication of our research group [33]. Clinical and demographic characteristics of sample 2 are provided in **Table 2**. This sample was now re-analyzed with respect to the predictive value of COR peak improvement during serial DEX/CRH tests for the therapeutic response. 40 drug-free patients suffering from a major depressive episode (DSM-IV criteria) were treated with either reboxetine (8 mg/day; n = 20) or mirtazapine (45 mg/day; n = 20) monotherapy for 5 weeks. Before, after 1 week and after 5 weeks of therapy, the dexamethasone/CRH-test was performed as described above and COR concentrations were measured. COR peak week 1 improvement was defined as lowering of the COR peak value between DEX/CRH test 1 (week 0 before treatment) and test 2 (after 1 week of treatment with either reboxetine or mirtazapine). COR peak week 5 improvement was established as a reduction of the COR peak level between test 1 (week 0) and test 3 (week 5). Likewise, COR basal week 1 or week 5 improvement was defined as lowering of the basal COR value at 03:00 PM (after administration of 1.5 mg DEX, but immediately before hCRH injection) between test 1 (week 0) and test 2 (week 1) or between test 1 (week 0) and test3 (week 5), respectively. In this sample, response was defined by a reduction of at least 50% in the 21-HAMD sum score after five weeks of treatment with either reboxetine or mirtazapine.

**Table 2:** Demographic and clinical parameters in 40 inpatients suffering from unipolar depression treated with either reboxetine (n = 20; 8 mg/day) or mirtazapine (n = 20; 45 mg/day) for 5 weeks (sample 1) [33].

	all patients	COR peak week 1	Statistics		COR peak week 5		Statistics
	(n = 40)	Im (n = 30)	NIm (n = 10)	COR peak week 1	Im (n = 24)	NIm (n = 16)	COR peak week 5
diagnoses							
MD, first episode	10	9	1	χ2 = 1.600; p = 0.206	7	8	χ2 = 0.556; p = 0.456
MD, recurrent	30	21	9		17	13	
gender (M/F)	17/23	14/16	3/7	χ2 = 0.853; p = 0.356	9/15	8/8	χ2 = 0.614; p = 0.433
age	47.9±14.6	49.7±14.3	42.5±15.1	F = 1.876; p = 0.179	49.6±14.2	45.4±15.3	F = 0.767; p = 0.387
height [cm]	170.1±9.2	169.6±8.3	171.8±11.9	F = 0.432; p = 0.515	169.2±8.7	171.4±10.2	F = 0.531; p = 0.470
BMI	25.0±4.1	24.9±4.2	25.3±4.2	F = 0.063; p = 0.803	25.1±4.2	24.8±4.2	F = 0.076; p = 0.784
age of onset	40.0±15.0	40.5±14.9	38.3±15.9	F = 0.158; p = 0.693	39.5±14.3	40.7±16.4	F = 0.063; p = 0.803
number of depressive episodes	2.58±1.74	2.63±1.92	2.40±1.08	F = 0.132; p = 0.718	2.50±1.93	2.69±1.45	F = 0.109; p = 0.743
duration of inpatient status	67.5±38.7	64.0±35.3	78.2±48.1	F = 1.013; p = 0.321	61.8±32.1	76.1±46.8	F = 1.306; p = 0.260
21-HAMD sum score at baseline	24.3±3.9	24.7±3.9	22.9±3.8	F = 1.594; p = 0.214	25.2±3.1	22.8±4.7	F = 3.818; p = 0.058

Mean±standard deviation (SD) is indicated. Patients are subdivided into COR week 1 Im (improvers)/COR peak NIm (nonimprovers) and into COR week 5 Im/NIm. COR peak week 1/week 5 Im ( = COR peak week 1/week 5 improver): patient with reduction of COR peak value in the DEX/CRH test after 1 week/5 weeks of treatment, as compared to baseline (week 0). MD = Major Depression. M = male, F = female. BMI = body mass index. 21-HAMD = Hamilton Depression Rating Scale, 21-item version. Statistics: results of χ^2^-test or oneway ANOVA are provided.

### Statistics

Demographic and clinical parameters were compared between suppressors and nonsuppressors (Heuser criterion) or between COR peak improvers and nonimprovers by the Pearson Chi-Square test for contingency tables or by Fisher's exact test with respect to qualitative variables and by one-way ANOVA for independent samples with regard to quantitative variables. Correlations between quantitative variables and endocrinological parameters were calculated using the rank order coefficient (Spearman's rho) since hormonal data were not normally distributed. Moreover, in sample 2 the baseline-corrected decrease in 21-HAMD sum scores during 5-week treatment was compared between COR peak week 1 improvers and nonimprovers and between COR peak week 5 improvers and nonimprovers using ANOVAs for repeated measurements. Thereby “time” (week 0–5) and “group” (improvers vs nonimprovers) were considered as within-subjects and between-subjects factors with six (“time”) and two (“group”) levels, respectively. Post-hoc tests with contrasts were additionally performed when “group” was among the significant influential factors. For the ANOVA procedures, a correction was applied to the F-value by means of adjusting the degrees of freedom by a factor Epsilon, if the sphericity test (Mauchly W test) was significant indicating a heterogeneity of covariances (Huyn-Feldt correction). In addition, Cramer's Phi was calculated in sample 2 for all patients and also separately in the reboxetine and the mirtazapine group in order to investigate putative associations between COR week 1 improvement/COR week 5 improvement and the clinical outcome after 5 weeks of treatment (response, remission).

As a nominal level of significance, alpha = 0.05 was accepted. The software program SPSS version 15.0 (SPSS Inc., Chicago, Illinois, USA) was used for data analysis.

The study was carried out according to the Declaration of Helsinki (http://www.wma.net) and had been approved by a local ethics committee (intramural review panel of the Ludwig-Maximilian-University of Munich, Faculty of Medicine).

## Results

**Figure 1:**
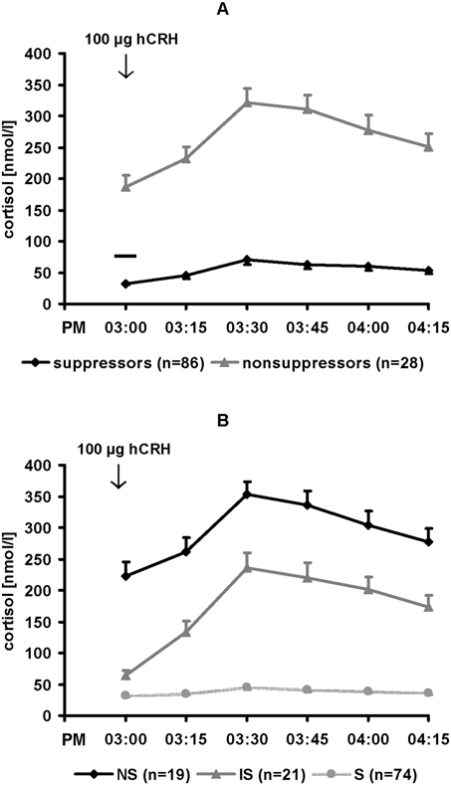
DEX/CRH test at admission in 114 depressed inpatients. (A) Subdivision into suppressors (n = 86) and nonsuppressors (n = 28) according to the Heuser criterion (nonsuppression: baseline COR≥75.3 nmol/l) as indicated by the cross bar. (B) Subdivision into nonsuppressors (NS; baseline COR≥50 ng/ml [∼137.95 nmol/l]; n = 19), intermediate suppressors (IS; baseline COR<50 ng/ml and peak COR≥50 ng/ml; n = 21), and suppressors (S; peak COR<50 ng/ml; n = 74) according to the Kunugi criterion. Baseline COR = COR at 03:00 PM. Mean+/−standard error of mean (SEM) is given.

Using a cut-off criterion of 75.3 nmol/l (COR level at 03:00 PM) for the definition of nonsuppression in the DEX/CRH test 1 at admission (Heuser criterion), in sample 1 only 28 out of 114 (∼24.6%) depressed inpatients were nonsuppressors whereas 86 (∼75.4%) acutely depressed patients were suppressors already before the beginning of antidepressant therapy (**Figure 1A**). Moreover, when also using the Kunugi criterion the suppressors (n = 74, i.e. 64.9%) were predominant as compared to nonsuppressors (n = 19 [∼16.7%]) or intermediate suppressors (n = 21 [∼18.4%]) (**Figure 1B**). With regard to the DEX/CRH test 1 suppressors and nonsuppressors (categorized by the Heuser criterion) did not differ in qualitative variables such as diagnoses, gender distribution, response or remission rates (p>0.05 in χ^2^-tests, respectively) (**Table 1**). Suppressors and nonsuppressors were also comparable in quantitative parameters such as age, height, BMI, age of onset, number of depressive episodes, and duration of inpatient status (p>0.05 in one-way ANOVA, respectively). However, nonsuppressors were prone to have higher 17-HAMD sum scores at baseline which was statistically significant (p<0.05) (**Table 1**). In addition, with respect to test 1 at admission severity of depressive symptoms (17-HAMD sum scores) was positively correlated with COR AUC values (Spearman's Rho = 0.238, p = 0.011).

**Figure 2:**
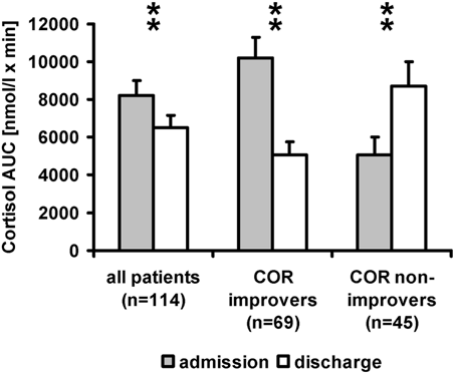
COR AUC values of DEX/CRH tests in 114 depressed inpatients at admission and at discharge. All patients (n = 114), and subgroups of COR improvers (n = 69) and of nonimprovers (n = 45) are shown. Mean+standard error of mean (SEM) is given. COR improver: reduction of COR peak value in the DEX/CRH test between admission and discharge. ** = highly significant differences in COR AUC values between admission and discharge (p<0.01).

The overall group of depressed inpatients (n = 114; sample 1) showed a significant decrease in COR AUC values during the DEX/CRH tests between admission and discharge (**Figure 2**). However, when the sample was subdivided in COR peak improvers (COR peak value test 1>COR peak value test 2; n = 69) and in COR peak nonimprovers (COR peak value test 1≤COR peak value test 2; n = 45), COR peak improvers displayed a marked reduction in COR AUC values during inpatient treatment whereas COR peak nonimprovers were characterized by a pronounced increase in COR AUC values in spite of clinical recovery and discharge (**Figure 2**). The same finding was observed if the patients were classified in patients receiving psychopharmacological drugs (n = 83) and patients treated with non-pharmacological treatment strategies such as TMS or ECT (n = 31). In the psychopharmacotherapy group, there were 52 COR peak improvers (mean COR AUC at admission: 9444.25±8606.81 nmol/l×min; mean COR AUC at discharge: 4599.75±4734.71 nmol/l×min) and 31 nonimprovers (mean COR AUC at admission: 4510.43±6065.80 nmol/l×min; mean COR AUC at discharge: 7790.39±7825.23 nmol/l×min). The non-pharmacological treatment group consisted of 17 COR peak improvers (mean COR AUC at admission: 12,531.01±10,626.87 nmol/l×min; mean COR AUC at discharge: 6442.82±8027.38 nmol/l×min) and 14 nonimprovers (mean COR AUC at admission: 6286.09±7256.49 nmol/l×min; mean COR AUC at discharge: 10,669.75±10,608.55 nmol/l×min). Considering the total sample (n = 114), COR peak improvers and nonimprovers were comparable with respect to diagnoses, gender distribution, response and remission rates (χ2-tests: p>0.05, respectively) (**Table 1**). Moreover, there were no significant differences between COR peak improvers and nonimprovers regarding age, height, BMI, number of depressive episodes or 17-HAMD sum score at baseline (oneway ANOVA: p>0.05, respectively). However, in COR peak nonimprovers a significantly earlier age of onset of the depressive illness and a significantly longer duration of time between admission and discharge (inpatient status) were found (p<0.05, respectively) (**Table 1**).

**Figure 3:**
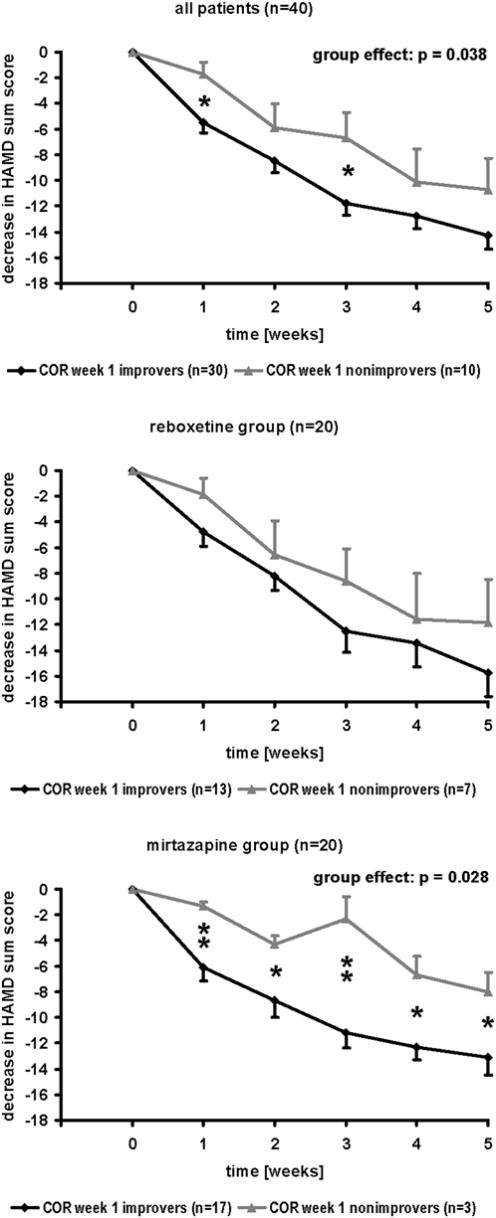
Analysis of COR week 1 improvers and nonimprovers. Mean value graphs of the decrease in 21-HAMD sum scores in 40 depressed patients treated with either reboxetine (n = 20) or mirtazapine (n = 20) for 5 weeks, subdivided into COR week 1 improvers and nonimprovers. COR week 1 improver = patient with reduction of COR peak value in the DEX/CRH test after 1 week of treatment, as compared to baseline (week 0). Mean+/−standard error of mean (SEM) is given. Significant group effects in the ANOVA for repeated measurements indicated. * = significant group differences in post-hoc test (p<0.05). ** = highly significant group differences in post-hoc test (p<0.01).

Demographic and clinical characteristics of sample 2 (40 depressed inpatients, treated with either reboxetine or mirtazapine for 5 weeks) are given in **Table 2**. There were no significant differences between COR week 1/week 5 improvers and nonimmprovers with regard to diagnoses, gender distribution, age, height, body mass index, age of onset, number of episodes, duration of total inpatient status, or severity of depression at baseline. COR peak week 1 improvement (reduction of the COR peak value in the DEX/CRH test after 1 week of treatment) was associated with alleviation of depressive symptoms. Regarding COR peak week 1 improvement in all patients (n = 40), repeated-measures ANOVA revealed a highly significant “time” effect, i.e. decrease in 21-HAMD sum scores (F = 50.173; d.f. = 2.642, 100.401; p<0.001). Moreover, a significant “group” effect was observed (F = 4.638; d.f. = 1, 38; p = 0.038) indicating a more pronounced amelioration of depressive symptoms in COR peak week 1 improvers than in nonimprovers (**Figure 3**). Post-hoc tests demonstrated significant differences between COR peak week 1 improvers and nonimprovers at week 1 and week 3 (p<0.05, respectively). No associations were found between COR peak week 1 improvement and response or remission rates when regarding all patients (Cramer's Phi: p>0.05, respectively; **Table 3**). It is also worth to be mentioned that 8 COR peak week 1 improvers were nonresponders at week 5, i.e. improvement of COR peak values after 1 week was not a guarantee (sufficient condition) for clinical response after 5 weeks. When analyzing separately depressed patients treated with reboxetine (n = 20) or mirtazapine (n = 20), significant “group” effects in the repeated-measures ANOVAs were obtained in the mirtazapine group (F = 5.738; d.f. = 1, 18; p = 0.028), but not in the reboxetine group (F = 1.410; d.f. = 1,18; p = 0.250) indicating better alleviation of depressive symptomatology in COR peak week 1 improvers treated with mirtazapine than in nonimprovers receiving this antidepressant. Moreover, in the mirtazapine group (Phi = 0.572; p = 0.010), but not in the reboxetine group (Phi = 0.121; p = 0.589) a significant association between COR peak week 1 improvement and response rate after 5 weeks of treatment was demonstrated (**Table 3**). Similar results were obtained if COR basal improvement after 1 week of treatment was used instead. Reduction of the basal COR value between test 1 and test 2 was significantly associated with better clinical response when regarding all patients, but also in separate analyses of the reboxetine and the mirtazapine groups (**Table 4**).

**Table 3:** COR peak week 1 improvement and COR peak week 5 improvement.

	response	statistics		remission	statistics	
	(NRs/Rs)	Cramer's phi	p-value	(NRm/Rm)	Cramer's phi	p-value
all patients (n = 40)	14/26			22/18		
COR peak week 1 Im (n = 30)	8/22			15/15		
		Phi = 0.303	**p = 0.056**		Phi = 0.174	p = 0.271
COR peak week 1 NIm (n = 10)	6/4			7/3		
COR peak week 5 Im (n = 24)	7/17			11/13		
		Phi = 0.150	p = 0.343		Phi = 0.226	p = 0.154
COR peak week 5 NIm (n = 16)	7/9			11/5		
reboxetine group (n = 20)	7/13			10/10		
COR peak week 1 Im (n = 13)	4/9			6/7		
		Phi = 0.121	p = 0.589		Phi = 0.105	p = 0.639
COR peak week 1 NIm (n = 7)	3/4			4/3		
COR peak week 5 Im (n = 14)	3/11			5/9		
		Phi = 0.435	**p = 0.052**		Phi = 0.436	**p = 0.051**
COR peak week 5 NIm (n = 6)	4/2			5/1		
mirtazapine group (n = 20)	7/13			12/8		
COR peak week 1 Im (n = 17)	4/13			9/8		
		Phi = 0.572	**p = 0.010**		Phi = 0.343	p = 0.125
COR peak week 1 NIm (n = 3)	3/0			3/0		
COR peak week 5 Im (n = 10)	4/6			6/4		
		Phi = 0.105	p = 0.639		Phi = 0.000	p = 1.000
COR peak week 5 NIm (n = 10)	3/7			6/4		

Response and remission rates after 5 weeks of treatment in 40 depressed patients (sample 2) treated with either reboxetine (n = 20) or mirtazapine (n = 20) [33]. Patients are subdivided into COR peak week 1 Im (improvers)/NIm (nonimprovers) and COR peak week 5 Im (improvers)/NIm (nonimprovers). COR peak week 1/week 5 Im ( = COR peak week 1/week 5 improver): patient with reduction of COR peak value in the DEX/CRH test after 1 week/5 weeks of treatment, as compared to baseline (week 0). NRs = nonresponders; Rs = responders. NRm = nonremitters; Rm = remitters. Statistics: Cramer's phi as measure of association for the chi-square test is provided. Significant results (p<0.05) or trends for significance (p<0.10) are given in bold letters.

**Table 4:** COR basal week 1 improvement and COR basal week 5 improvement.

	response	statistics		remission	statistics	
	(NRs/Rs)	Cramer's phi	p-value	(NRm/Rm)	Cramer's phi	p-value
all patients (n = 40)	14/26			22/18		
COR basal week 1 Im (n = 28)	5/23			12/16		
		Phi = 0.549	**p = 0.001**		Phi = 0.373	**p = 0.018**
COR basal week 1 NIm (n = 12)	9/3			10/2		
COR basal week 5 Im (n = 24)	6/18			11/13		
		Phi = 0.257	p = 0.104		Phi = 0.226	p = 0.154
COR basal week 5 NIm (n = 16)	8/8			11/5		
reboxetine group (n = 20)	7/13			10/10		
COR basal week 1 Im (n = 13)	2/11			5/8		
		Phi = 0.560	**p = 0.012**		Phi = 0.314	p = 0.160
COR basal week 1 NIm (n = 7)	5/2			5/2		
COR basal week 5 Im (n = 14)	2/12			5/9		
		Phi = 0.663	**p = 0.003**		Phi = 0.436	**p = 0.051**
COR basal week 5 NIm (n = 6)	5/1			5/1		
mirtazapine group (n = 20)	7/13			12/8		
COR basal week 1 Im (n = 15)	3/12			7/8		
		Phi = 0.545	**p = 0.015**		Phi = 0.471	**p = 0.035**
COR basal week 1 NIm (n = 5)	4/1			5/0		
COR basal week 5 Im (n = 10)	4/6			6/4		
		Phi = 0.105	p = 0.639		Phi = 0.000	p = 1.000
COR basal week 5 NIm (n = 10)	3/7			6/4		

Response and remission rates after 5 weeks of treatment in 40 depressed patients (sample 2) treated with either reboxetine (n = 20) or mirtazapine (n = 20) [33]. Patients are subdivided into COR basal week 1 Im (improvers)/NIm (nonimprovers) and COR basal week 5 Im (improvers)/NIm (nonimprovers). COR basal week 1/week 5 Im ( = COR basal week 1/week 5 improver): patient with reduction of COR basal value in the DEX/CRH test after 1 week/5 weeks of treatment, as compared to baseline (week 0). NRs = nonresponders; Rs = responders. NRm = nonremitters; Rm = remitters. Statistics: Cramer's phi as measure of association for the chi-square test is provided. Significant results (p<0.05) or trends for significance (p<0.10) are given in bold letters.

**Figure 4:**
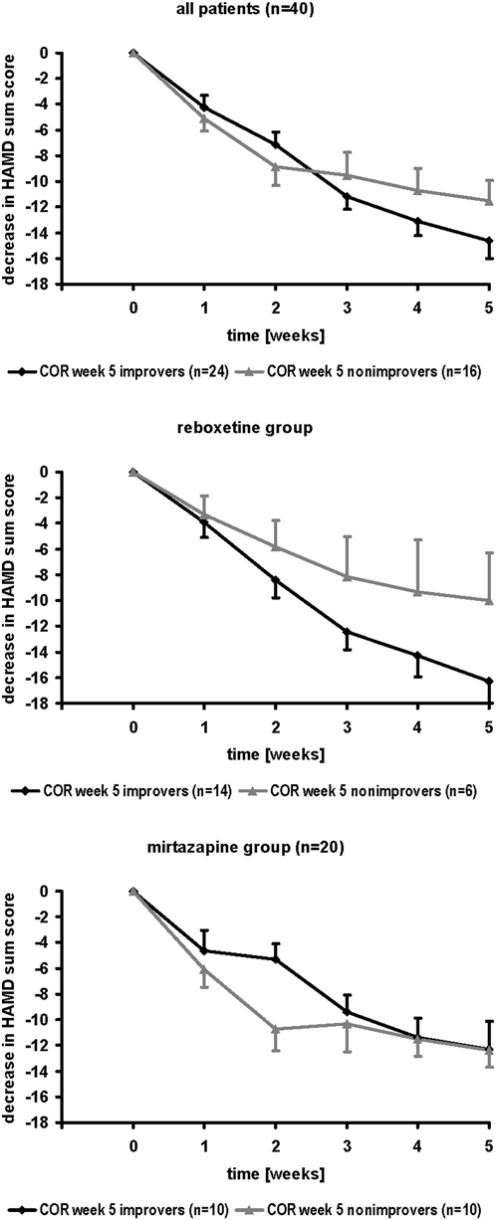
Analysis of COR week 5 improvers and nonimprovers. Mean value graphs of the decrease in 21-HAMD sum scores in 40 depressed patients treated with either reboxetine (n = 20) or mirtazapine (n = 20) for 5 weeks, subdivided into COR week 5 improvers and nonimprovers. COR week 5 improver = patient with reduction of COR peak value in the DEX/CRH test after 5 weeks of treatment, as compared to baseline (week 0). Mean+/−standard error of mean (SEM) is given.

Analyzing putative associations between the attenuation of COR peak values at week 5 and antidepressant efficacy in the whole sample (n = 40), there was no significant “group” effect in the repeated-measures ANOVA between COR peak week 5 improvers and nonimprovers (F = 0.358; d.f. = 1, 38; p = 0.553) (**Figure 4**). In addition, when analyzing separately for reboxetine and mirtazapine, no significant group effects were seen in the repeated-measures ANOVA between COR peak week 5 improvers and nonimprovers either (reboxetine: F = 2.314; d.f. = 1, 18; p = 0.146; mirtazapine: F = 0.692; d.f. = 1, 18; p = 0.416). However, when using Cramer's Phi as effect size parameter, relevant associations between COR week 5 improvement and clinical outcome (response rate, remission rate) could be shown for the reboxetine group which were nearly significant (response rate: Phi = 0.435; p = 0.052; remission rate: Phi = 0.546; p = 0.051), whereas no such association were seen in the mirtazapine group (**Table 3**).

Using COR basal improvement after 5 weeks of treatment as parameter for changes in HPA axis activity, the analysis revealed a significant association between COR basal week 5 improvement and response/remission in the reboxetine group, but not in the mirtazapine group (**Table 4**).

## Discussion

One of our main results is the finding that in acutely depressed inpatients the nonsuppression rate in the DEX/CRH test at admission was 24.6% (28 out of 114) according to the Heuser criterion which focuses on the 1.5 mg dexamethasone suppression status and does not consider the CRH-stimulated COR concentrations [25]. When using the Kunugi criterion which also involves the COR levels after CRH challenge [52], [53], the rates of nonsuppression (16.7%) or intermediate suppression (18.4%) were somewhat higher if added together (35.1%). However, in any case the proportion of acutely and severely depressed inpatients who were identified by nonsuppression in the DEX/CRH test was much lower in our study than originally expected [25]. In a large meta-analysis [55] including more than 5,000 depressed patients, a sensitivity of the single dexamethasone suppression test (DST) of 44% was found using the “Carroll criterion” [56] (nonsuppression in the DST: COR level >5 µg/dl the day after oral administration of 1 mg dexamethasone). In the original study of the Max-Planck-Institute of Psychiatry in Munich, introducing the combined DEX/CRH test in the literature, it was reported that the sensitivity of the DEX/CRH test in depression is about 80 to 90% if the control subjects are matched for age and gender and thus greatly exceeds the sensitivity of the standard DST. Moreover, a dichotomous cut-off criterion of 40 ng/ml (110 nmol/l) for the baseline COR concentration in the DEX/CRH test (1.5 mg dexamethasone suppression status) was proposed to differentiate between suppressors and nonsuppressors [25], [57]. However, in a cross-laboratory validation of the Max-Planck-Institute it was discovered that the RIA analysis of plasma COR which had been performed produced concentration values with a linear bias [35]. Since the biased COR levels were elevated by a factor of 1.46 in comparison with those at other laboratories, the cut-off criterion was corrected accordingly and was now proposed as 27.5 ng/ml (75.3 nmol/l) [32], [51].

Even if the corrected cut-off criterion (75.3 nmol/l) is used, in more recent studies the sensitivity (nonsuppression rate) in acutely depressed inpatients using the DEX/CRH test is surprisingly low, possibly lower than the sensitivity of the standard DST (44%), and amounts to 31.6% (12 out of 38 [32]), 16.6% (35 out of 211 [51]), or 24.6% (28 out of 114; present study). Moreover, when using the Kunugi criterion (a) nonsuppression: baseline COR≥50 ng/ml [∼137.95 nmol/l]) b) intermediate suppression: baseline COR<50 ng/ml and peak COR≥50 ng/ml c) suppression: peak COR<50 ng/ml) which considers also the CRH effects within the combined DEX/CRH test, the rates of nonsuppression or intermediate suppression in this test (35.1% in our sample) is lower than expected. In the original investigation of Heuser and coworkers [25] a sensitivity of 80 to 90% in the DEX/CRH test was only reached if depressed patients and control subjects were clustered into different age ranges and highly sophisticated statistical methods such as multivariate analysis of variance or discriminant analysis were used. However, these analyses are not practicable in the clinical situation which requires clear dichotomous variables to differentiate between suppression and nonsuppression. No study has been performed so far confirming the originally reported high sensitivity of the DEX/CRH test of more than 80% by using a criterion which is applicable under clinical conditions. Apparently an ideal cut-off criterion has not been established yet for the DEX/CRH test.

Nevertheless, a considerable part of acutely depressed patients shows normally regulated HPA axis activity in the DEX/CRH test already before antidepressant treatment and may though benefit from this therapy. In fact the severity of depression was significantly higher in baseline nonsuppressors than in suppressors in our study and a significant positive correlation between baseline 21-HAMD sum score and COR AUC values (test 1) could be demonstrated in our investigation as it has been reported in previous studies [35], [53], [58], [59]. However, the response and remission rates in nonsuppressors and suppressors on test 1 were comparable. Therefore, a single DEX/CRH test performed within the first week after admission is obviously not suitable for prediction of the acute treatment response.

The best predictor for acute antidepressant efficacy seems to be the responsiveness of the HPA system and the change of DEX/CRH test results within the first one or two weeks of antidepressant treatment. In our investigation, attenuation of HPA axis activity (reduction of COR basal value, reduction of COR peak value) in the whole patient sample after one week of pharmacotherapy was significantly associated with the subsequent alleviation of depressive symptoms. This is in line with studies reported by Ising and colleagues who found improved HPA system regulation in a second DEX/CRH test (performed 2 or 3 weeks after the first test at the beginning of the study) to be associated with beneficial treatment response after 5 weeks [51], [54]. Thus, performance of two DEX/CRH tests in acutely depressed patients with an interval of 1 up to 3 weeks seems to be a potential biomarker with certain significance for the subsequent therapeutic response.

Since improvement of basal COR, representing a single dexamethasone suppression test (DST), was at least as powerful in prediction of therapeutic response as improvement of the COR peak value (as part of the combined DEX suppression/CRH stimulation test), one may assume that performance of 2 subsequent dexamethasone suppression tests (DST) may be a feasible and appropriate predictor of clinical outcome. One single DST prior to treatment does not reliably predict response to antidepressant therapy [60]. However, reduction of COR in 2 subsequent DST is of predictive value (our data). As it has been shown by Carroll and colleagues 4 PM and 11 PM samples are much better to detect COR nonsuppression (as defined by the 5 µg/dl-criterion in the standard DST) than 8 AM samples [61]. Furthermore, the same research group could demonstrate that with the 1-mg DEX dose the sensitivity of the DST greatly exceeds that of the 2-mg DST [61]. Thus, performance of 2 subsequent 1-mg DST using 4 PM or 11 PM samples [61] or two subsequent 1.5 mg DST using 3 PM samples (our study) may be an easy and appropriate way to predict therapeutic response. Our results are confirmed by former studies performing serial DST and suggesting that downregulation of the HPA system activity as measured by the DST precedes or coincides with the amelioration of depressive symptoms [42], [62]–[65].

However, there seem to be differences between antidepressant drugs which are related to their distinct biochemical properties. Reboxetine is a norepinephrine reuptake inhibitor which acutely stimulates COR secretion [66] whereas mirtazapine does not cause reuptake inhibition but is an antagonist at α_2_-, 5-HT_2_-, 5-HT_3_-, and histamine H_1_ receptors and acutely inhibits COR secretion [67], [68]. Apparently, early change of HPA axis activity (week 1) induced by mirtazapine is related to clinical outcome after 5 weeks whereas 5-week response to reboxetine is associated with late change in DEX/CRH test results at week 5 (**Table 2**, **Figure 3**, **Figure 4**). Reuptake inhibiting antidepressants such as reboxetine, selective serotonin reuptake inhibitors (SSRIs) or tricyclic antidepressants acutely stimulate cortisol and ACTH secretion both in healthy subjects [69] and in depressed patients [70], [71] after single administration and may gradually normalize HPA axis hyperactivity in depressed patients if they are given daily for several weeks via up-regulation of mineralocorticoid receptor and glucocorticoid receptor mRNA levels [44], [46], [72], [73], down-regulation of pro-opiomelanocortin mRNA expression in the pituitary gland [74], and decrease of CRH gene expression and CRH mRNA synthesis in the paraventricular nucleus [75], [76] thereby enhancing mineralocorticoid receptor and glucocorticoid receptor function and restoring the disturbed feedback control. On the contrary, mirtazapine rapidly reduces HPA axis hyperactivity in depressed patients within one week which can be explained most likely by direct pharmacoendocrinological effects of mirtazapine such as antagonism at central 5-HT_2_- and H_1_-receptors thereby inhibiting the hypothalamic CRH release. After 5 weeks of mirtazapine therapy in depressed patients, there is a partial “rebound” phenomenon which can probably be explained by a compensatory up-regulation of CRH receptors at the pituitary gland during several weeks of mirtazapine treatment leading to a partial re-enhancement of cortisol and ACTH output after exogenous administration of 100 µg hCRH during the DEX/CRH test [33]. Obviously, these different effects on the time course of HPA axis activity in depressed patients during reboxetine or mirtazapine treatment are also reflected by diverse associations with clinical outcome (response related to early changes in HPA axis activity during mirtazapine treatment and to late changes in HPA system during reboxetine therapy).

However, two limiting issues have to be pointed out in this context: First, an early improvement of HPA axis hyperactivity (e.g. within 1 week of treatment) is not necessarily followed by a favourable response and therefore is not a sufficient condition for a beneficial treatment outcome. In the present study, 8 out of 40 depressed patients were classified as COR peak week 1 improvers but were nonresponders after 5-week treatment with either reboxetine or mirtazapine. Moreover, in a former study of our research group, mirtazapine effectively reduced the overshoot of COR during the DEX/CRH test within 1 week of treatment in 40 depressed inpatients, but this attenuation of HPA axis activity occurred both in 5-week responders and nonresponders and was not related to clinical improvement [77]. Therefore, the importance of an early improvement of HPA axis dysregulation for the prediction of the acute antidepressant response is limited. Second, the association between clinical response to the norepinephrine reuptake inhibitor reboxetine and late changes in HPA system activity (week 5) in our investigation is not confirmed by other clinical trials investigating the impact of reuptake inhibiting antidepressants on HPA axis function in depression, since the decrease in COR levels during serial DEX/CRH tests after 4 to 6 weeks of pharmacotherapy has been found to be comparable in responders and nonresponders in these studies [26]–[33].

Moreover, it is remarkable in our study that a considerable proportion of depressed inpatients (39.5%, i.e. 45 out of 114) showed a pronounced enhancement of HPA axis activity shortly before discharge in spite of clinical recovery. Our finding is supported by other researchers who also found an enhanced HPA system activity at discharge in a notable part of depressed patients [34], [35], e.g. in 21 out of 74 (28.4%) investigated patients [35]. It is important to note that in our study HPA system nonimprovers at discharge were prone to have an earlier age of onset of the depressive illness and a longer duration of the inpatient stay as compared to improvers. Furthermore it is known that HPA axis activity at discharge in spite of clinical improvement is associated with a higher risk for relapse of depression with regard to medium-term or long-term outcome [34]–[37], which has not been investigated in the present study. Nevertheless attenuation of HPA axis activity during antidepressant therapy is obviously not a necessary condition for acute clinical recovery.

Taken together, it can be concluded from our data that the sensitivity (rate of nonsuppression) of the combined DEX/CRH test in acutely depressed patients is much lower than originally reported. Moreover, the performance of a single DEX/CRH test shortly after admission does not predict the therapeutic response. The best predictor for response seems to be the early responsiveness and downregulation of HPA axis activity within the first 1 or 2 weeks of antidepressant treatment as measured by 2 subsequent DEX/CRH tests. Possibly, the performance of 2 subsequent standard DST may be of comparable predictive value and can be offered to depressed patients more easily in the clinical situation. However, the significance of these potential biomarkers is limited since early improvement of HPA axis dysregulation is not necessarily followed by a favourable therapeutic response and is therefore not a sufficient condition for a beneficial treatment outcome. After 4–6 weeks of antidepressant treatment, the attenuation of HPA axis activity is comparable in responders and nonresponders in most studies although an association between COR week 5 improvement and clinical response to reboxetine could be demonstrated in the present investigation. At discharge, a substantial part of depressive patients show even an enhancement of HPA axis activity in spite of clinical recovery. Thus, downregulation of HPA system function is not a necessary condition for clinical improvement. However, patients with persistence of HPA axis hyperactivity at discharge are known to have a higher risk for relapse during the following 6 months. Our data underline the importance of HPA axis dysregulation for treatment outcome in major depression, although restoration of HPA system dysfunction seems to be neither a necessary nor a sufficient determinant for acute treatment response.
